# Research methods for subgrouping low back pain

**DOI:** 10.1186/1471-2288-10-62

**Published:** 2010-07-03

**Authors:** Peter Kent, Jennifer L Keating, Charlotte Leboeuf-Yde

**Affiliations:** 1 Department of Physiotherapy, Monash University, Melbourne, Australia; 2 Research Department, Spine Centre of Southern Denmark, Lillibaelt Hospital, Middelfart, Denmark; 3 Institute of Regional Health Services Research, University of Southern Denmark, Odense, Denmark

## Abstract

**Background:**

There is considerable clinician and researcher interest in whether the outcomes for patients with low back pain, and the efficiency of the health systems that treat them, can be improved by 'subgrouping research'. Subgrouping research seeks to identify subgroups of people who have clinically important distinctions in their treatment needs or prognoses. Due to a proliferation of research methods and variability in how subgrouping results are interpreted, it is timely to open discussion regarding a conceptual framework for the research designs and statistical methods available for subgrouping studies (a method framework). The aims of this debate article are: (1) to present a method framework to inform the design and evaluation of subgrouping research in low back pain, (2) to describe method options when investigating prognostic effects or subgroup treatment effects, and (3) to discuss the strengths and limitations of research methods suitable for the hypothesis-setting phase of subgroup studies.

**Discussion:**

The proposed method framework proposes six phases for studies of subgroups: studies of assessment methods, hypothesis-setting studies, hypothesis-testing studies, narrow validation studies, broad validation studies, and impact analysis studies. This framework extends and relabels a classification system previously proposed by McGinn et al (2000) as suitable for studies of clinical prediction rules. This extended classification, and its descriptive terms, explicitly anchor research findings to the type of evidence each provides. The inclusive nature of the framework invites appropriate consideration of the results of diverse research designs. Method pathways are described for studies designed to test and quantify prognostic effects or subgroup treatment effects, and examples are discussed. The proposed method framework is presented as a roadmap for conversation amongst researchers and clinicians who plan, stage and perform subgrouping research.

**Summary:**

This article proposes a research method framework for studies of subgroups in low back pain. Research designs and statistical methods appropriate for sequential phases in this research are discussed, with an emphasis on those suitable for hypothesis-setting studies of subgroups of people seeking care.

## Background

Several authors [1,2] have argued that low back pain is most accurately classified as pain associated with serious pathology, pain associated with nerve compression, or non-specific low back pain (NSLBP). Under this approach, approximately 80% of low back pain in primary care is classified as NSLBP [1] and investigations into treatment efficacy for this condition have identified only moderate treatment effects.

However, most clinicians [3,4] and researchers [3] believe NSLBP to be a number of conditions, and subgrouping NSLBP is currently of clinical and research interest [5-10]. This interest is premised on the notion that patient outcomes might be improved with more precise targeting of treatment, and health system efficiency might be improved with more effective triage of patients.

Many NSLBP subgrouping systems have been proposed. Some aim to identify people whose pain is associated with a particular pathoanatomical condition, based on their presenting symptoms and signs (diagnostic subgroups) [11,12]. Other systems aim to identify people likely to respond favourably to particular treatment regimens (treatment effect modifier subgroups) [13-15], while other systems aim to identify people with particular prognoses (prognostic factor subgroups) - such as those at risk of chronicity [10]. While there is no shortage of opinions about the composition of clinically important NSLBP subgroups, there is very little consensus regarding the symptoms and signs that identify these subgroups [16].

Subgrouping studies have previously been classified into three broad stages of research: exploratory studies that seek to identify subgroups, studies that attempt to validate subgroups and studies that test the capacity of subgrouping to positively influence routine clinical care [17]. The research designs and statistical methods appropriate for subgrouping studies vary depending on whether the aim of the subgrouping is prognostic, therapeutic or diagnostic, and also vary depending on the stage of the research.

Subgrouping research is fraught with methodological pitfalls and many authors have described reasons for caution in the conduct, interpretation and reporting of such studies [18-22]. In this context, there has been a proliferation of subgrouping studies in NSLBP, most of which have been hypothesis-setting and they report highly variable methods. Even among studies that report similar methods, their authors may have different opinions about the level of evidence these studies are capable of providing. Therefore, in subgrouping research not only is methodological rigor very important but there is also a need for an accepted method framework in which to classify, evaluate and discuss this research with a common vocabulary.

The aims of this debate article are to present a method framework for conducting and evaluating subgrouping research in low back pain, and to discuss the strengths and limitations of research methods suitable for hypothesis-setting studies. The focus of the article is on research method and where appropriate, examples of studies are used to illustrate concepts. However, this article is not a review of the findings of subgrouping research and other examples of studies may have been equally appropriate.

## Discussion

### Prognostic factors, treatment effect modifiers and clinical prediction rules

In this proposal we adopt earlier recommendations in distinguishing between prognostic factors and treatment effect modifiers [23,24]. Prognostic factors are symptoms, signs or other characteristics that indicate likely outcomes regardless of treatment. Treatment effect modifiers are symptoms, signs or characteristics that indicate likely response to a specific treatment (a subgroup treatment effect). This distinction has important implications for the methods suitable for research of subgroups. Patient outcomes are usually the product of a combination of treatment effects and prognostic factor effects, unless the treatment is completely ineffective. Therefore, studies need to use particular designs if these effects are to be teased apart. It has been reported that this distinction is commonly misunderstood [23].

#### Prognostic factors

The effect of prognostic factors can be studied in data from cohort studies ('single-group' designs) of usual care. Usual care implies that treatments are various, uncontrolled and reflective of common practice. Under these circumstances, it is assumed that the heterogeneity of treatments washes out specific treatment modifier effects. In contrast and at the other extreme, when predictors of outcome are investigated in cohort studies in which all participants receive only one treatment, it is not possible to differentiate between which factors predictive of outcome are prognostic factors and which are effect modifiers specific to that treatment [23].

Prognostic factors have also been studied using data from randomised controlled trials ('two-group' designs). One approach is to study predictive factors only in a control group that received either placebo care or usual care. Conceptually, this is similar to a prospective cohort study. Another approach is to study (as a single group) the whole cohort from a trial that showed no differences in outcome for the experimental and the control treatments. Where the control treatment was not placebo or no treatment, this latter approach is problematic if the treatments had a clinical effect, as the predictive factors may contain treatment effect modifiers common to both treatments. The generalisability of findings from studies of prognostic factors are always limited by the selection criteria of the study, and clinical trials tend to have more restrictive inclusion criteria than cohort studies. Contemporary summaries are available of key methodological issues for cohort studies of prognostic factors [18,20,25,26].

#### Treatment effect modifiers

In contrast, the precise measurement of treatment effect modifiers requires data from randomised controlled trials [27]. The appropriate trial design varies depending on the type of research question being investigated. As precise identification of the presence of treatment effect modification requires a test of subgroup/treatment effect interaction, currently only two designs for controlled trials are well suited for measuring treatment modifier effects [23,28,29]. Examples of such studies are those by Childs et al (2004) [14] and Brennan et al (2006) [13]. There are a number of concise summaries available for readers who are seeking greater detail on methodological issues for randomised controlled trials in which treatment effect subgroup analysis is planned [19,21-24,30].

#### Clinical prediction rules

Whether used in the investigation of prognostic factors or treatment effect modifiers, many statistical techniques produce measures of association that can be difficult for clinicians to apply to individual patients. In response to this, clinical prediction rules are increasingly being used as a means to express the likely response of a subgroup in clinically interpretable ways. They also allow the accuracy of this predictive capacity to be described [31]. A method for forming a prediction rule is detailed in Additional File 1.

### Method framework overview

**Figure 1:**
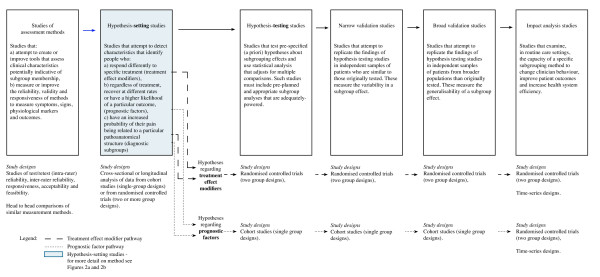
Conceptual phases of research into subgroups

The proposed method framework classifies subgrouping studies into six phases of research: studies of assessment methods, hypothesis-setting studies, hypothesis-testing studies, narrow validation studies, broad validation studies, and impact analysis studies. These are defined in Figure 1. This framework extends descriptive terms previously suggested by McGinn et al (2000) [17] as suitable for classifying studies of clinical prediction rules. It does so by adding an initial phase of 'studies of assessment methods', and by splitting the process described by McGinn as 'derivation studies' into two phases: hypothesis-setting studies and hypothesis-testing studies.

Extending and modifying these phases in the proposed framework was undertaken to achieve a number of purposes. The first purpose was to allow categorisation of an increasing number of studies that seek to devise measures of subgroup-specific characteristics, especially measures of physical impairment. The second purpose was to better describe the quality of evidence provided by studies in the pre-validation phases, in recognition that authors were interpreting that quality in different and contradictory ways. The third purpose was to broaden the framework to include subgrouping studies that do not express findings using clinical prediction rules.

#### Phases of research into subgroups

##### Studies of assessment methods

Within the proposed method framework, the first phase in subgrouping research comprises studies that attempt to create or improve tools that assess clinical characteristics potentially indicative of subgroup membership, or to determine the measurement properties (clinimetrics) of those tools. Guidance is available on suitable research designs and statistical methods to perform such studies [32-36]. An example of a study of a novel tool for assessing potential subgroup membership is Ferreira et al (2004)[37], which investigated an ultrasound test to measure, in clinical settings, the automatic recruitment of trunk muscles in people with low back pain.

##### Hypothesis-setting studies

The second phase (hypothesis-setting) is represented by studies that attempt to determine which characteristics identify people in clinically important subgroups, and the magnitude of any prognostic effects or treatment effect modification attributable to these subgroups. Other authors have argued that treatment effect modifiers can only be determined in randomised controlled trials[23] and that the probability (p value) of treatment responses associated with specific subgroups should be adjusted to reflect multiple statistical comparisons[24]. We suggest that within a hypothesis-generating phase, these criteria can be relaxed and instead applied later during rigorous hypothesis-testing studies. For example, we suggest that *during this exploratory phase*, data from cohort studies may generate useful hypotheses about potential treatment effect modifiers (for example Flynn et al 2004), and that it is permissible to perform post-hoc multiple comparisons without Bonferroni-type corrections.

##### Hypothesis-testing studies

The third phase (hypothesis-testing) in subgrouping research comprises studies that test pre-specified (a priori) hypotheses about subgrouping effects in samples of people independent from but similar to those people who participated in the hypothesis-setting phase. *During this confirmatory phase *there is a need for the rigorous testing of only pre-specified hypotheses and for appropriate statistical adjustment for multiple comparisons. We believe that this distinction between hypothesis-setting and hypothesis-testing studies would reconcile differences in interpretation as to the quality of evidence of subgroup effect that particular studies provide.

Replication of prognostic effects or treatment effect modifion in an independent sample under stringent research conditions is the central aim of hypothesis-testing studies and is a method of external validation. The chance of spurious, sample-specific effects or associations in hypothesis-setting studies is so high in subgrouping research, that Rothwell (2005) [19] suggests that the best test of the validity of subgroups is not significance testing but replication in an independent sample. Quasi-replication within the hypothesis-setting stage by use of iterative statistical techniques (such as boot-strapping) is an inadequate substitute for replication in an independent sample [17,38]. This is because the repeated testing of findings on sub-samples of the original data only partially counters problems associated with sample-specific relationships between predictors and outcomes, as they are test a relationship in the cohort in which the relationship was first established.

##### Narrow validation studies

The fourth phase (narrow validation) comprises studies that attempt to validate the findings of hypothesis-testing studies in samples of people who are independent from, but similar to, those who participated in the hypothesis-testing phase. Such studies provide insight into the variability of a subgroup effect in the target population [27].

##### Broad validation studies

The fifth phase (broad validation) comprises studies that test the findings of hypothesis-testing studies in samples of people who differ from those who previously participated. The clinical characteristics of these samples of patients may differ on dimensions such as the spectrum of the disorder, demographic and psychosocial profile, culture and language, co-morbidities and care settings (primary/secondary/tertiary care). Similarly, the experience, training and professional discipline of the clinicians may vary from those providing care in earlier studies. Broad validation establishes the generalisability of the subgroup findings beyond the clinical and professional profile of the people originally studied [17].

##### Impact analysis studies

The sixth and last phase (impact analysis) comprises studies that seek to establish the feasibility of uptake of the subgrouping scheme in practice and the capacity of subgrouping to improve outcomes in routine clinical care. Subgroups that have been shown to have predictive capacity in hypothesis-testing and validation studies may still not be effectively implemented in routine care, due to issues such as perceived importance by clinicians, patient-perceived acceptability and the practicality of assessing predictor variables [26,39].

### Methods for performing hypothesis-setting studies of subgrouping

Most of the subgrouping studies in low back pain have been hypothesis-setting and they have used highly variable research designs and statistical methods. We have classified these designs and methods into three categories. The purpose of the categories is to clarify suitable method pathways in hypothesis-setting studies, although it is possible that studies may exist that contain elements from more than one pathway.

#### Subgroups based on opinion (clinical observation)

The first of these categories is opinion-based subgroups that originate from clinical observation. An example is the McKenzie subgroup of patients who display a directional preference [40], which was initially based on an astute clinical observation that some people display pain that responds to particular movements.

#### Subgroups based on physiological/psychosocial models that are derived from experimental observation

The second category is subgroups based on physiological/psychosocial constructs that are derived from experimental observation. An example is O'Sullivan's 'Mechanism-based classification' [41].

**Figure 2:**
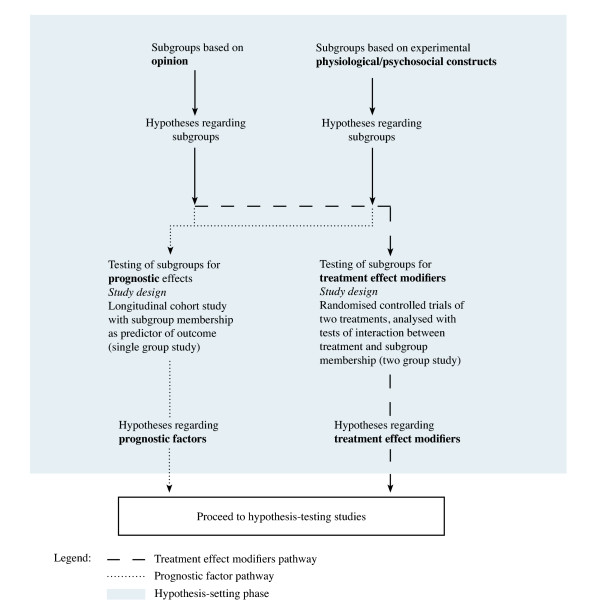
Flowchart for hypothesis-setting studies of subgroups based on opinion or based on physiological models

We argue that within the hypothesis-setting phase, opinion-based subgroups and subgroups based on experimental constructs need to be formally tested for treatment modification effects using appropriately designed randomised controlled trials, and/or tested for prognostic effects using a cohort study design. Method pathways for opinion-based subgroups and subgroups based on physiological/psychosocial experimental constructs are shown in Figure 2.

#### 'Data-driven' subgroups based on statistical analysis

**Figure 3:**
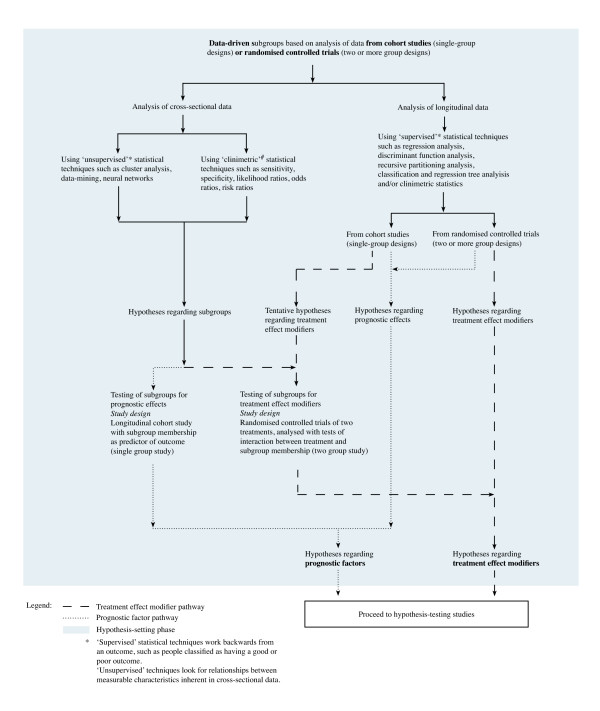
Flowchart for hypothesis-setting studies of subgroups based on 'data-driven' analysis

The third category of designs and methods used in hypothesis-setting studies is 'data-driven' subgroup analysis, where data from cohort studies or randomised controlled trials are investigated using cross-sectional statistical analysis, or investigated using longitudinal statistical analysis. These forms of analysis are called 'data-driven' because a subgroup is being formed retrospectively (post-hoc) from the characteristics of the sample data [22], rather than on clinical observation or a physiological/psychosocial experimental model. Method pathways for data-driven subgroups are shown in Figure 3.

Data-driven subgroups can be identified in two ways: either in relation to an outcome or by identifying variables that are associated with each other without regard to an outcome. Statisticians call these two main classes of statistical approaches 'supervised' techniques and 'unsupervised' techniques respectively. Both have methodological advantages and disadvantages. The first main class of these statistical techniques are called 'supervised' because these techniques work backwards from an outcome in longitudinal data, such as people classified as responders or non-responders to a treatment regimen. Examples of supervised statistical techniques include regression analysis, discriminant function analysis, recursive partitioning analysis, and classification and regression trees. The other main class of statistical techniques used in data-driven subgrouping is called 'unsupervised' because these techniques do not work backwards from an outcome but instead look for relationships between measurable characteristics inherent in cross-sectional data. Examples of unsupervised statistical techniques include cluster analysis, data-mining and neural networks.

A sub-class of statistical techniques are known by statisticians as 'clinimetric' techniques. These techniques include sensitivity, specificity, likelihood ratios, odds ratios, risk ratios and pre- and post-test probability. In hypothesis-setting studies of subgroups of low back pain, one set of circumstances where these statistical techniques have been used is with cross-sectional data. An example is when seeking to identify the clinical characteristics of people who respond to 'diagnostic' injections ('diagnostic' subgroups). Another circumstance where these statistical techniques have been used is within the formation of clinical prediction rules. For example, where subgroup characteristics have been identified using other methods, they have been used to determine the optimal combination of predictor variables that provides the greatest classification accuracy.

#### Data-driven subgroups from the analysis of longitudinal data - using 'supervised' statistical techniques

**Figure 4:**
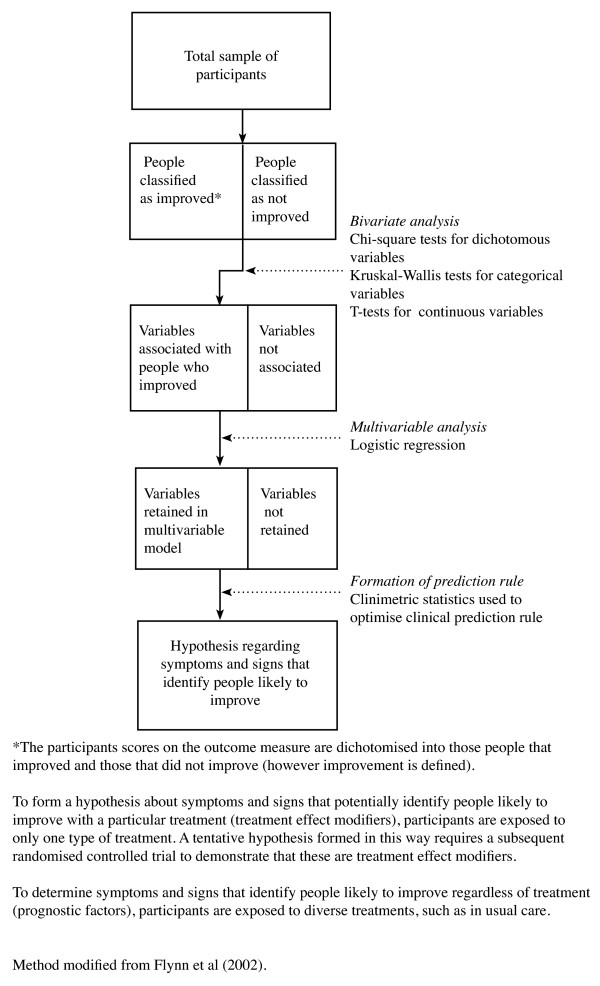
Example of the use of a 'supervised' statistical technique (logistic regression) on longitudinal data in a hypothesis-setting cohort study (single-group design).

In hypothesis-setting subgrouping analysis of longitudinal data, the most common data-driven approach is the use of 'supervised' statistical techniques. An example of such a study is the formation of the Flynn manipulation prediction rule [42]. In this cohort study, a group of people who all received the same treatment (spinal manipulation and range-of-motion exercises) was investigated with the aim of constructing a clinical prediction rule capable of identifying people likely to improve with this treatment. Logistic regression was used to determine which symptoms and signs were predictive of people who improved, and clinimetric statistics were used to determine what combination of those symptoms and signs provided the greatest predictive capacity (Figure 4). Being a cohort study, hypotheses formed about treatment effect modifiers could only be tentative, as such a study design cannot clearly differentiate between treatment effect modifiers and prognostic factors.

**Figure 5:**
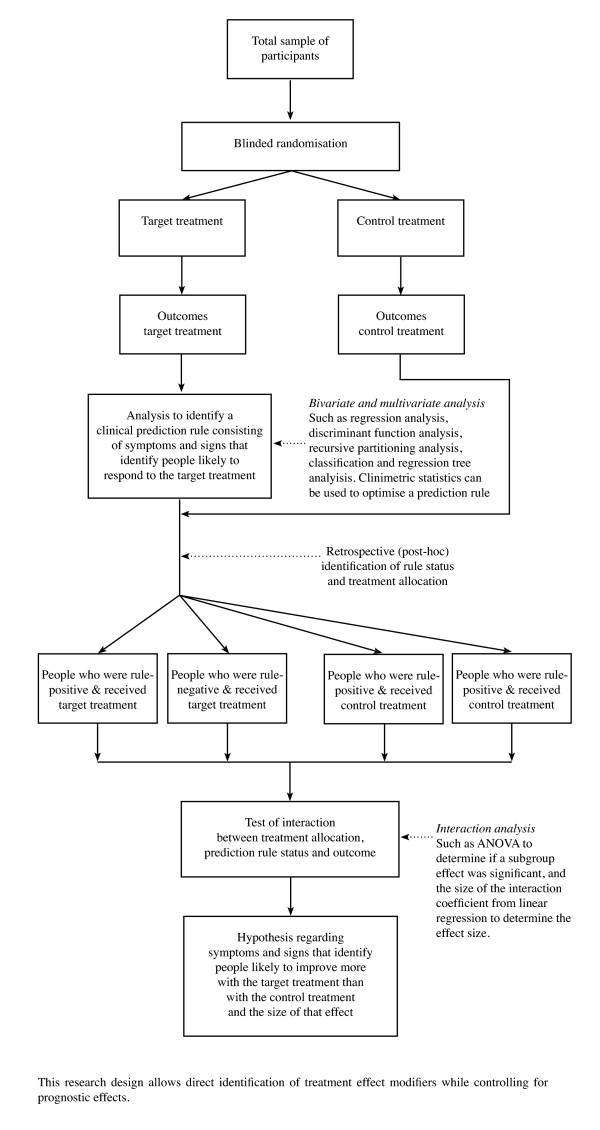
**Example of the use of 'supervised' statistical techniques (such as regression and ANOVA) on longitudinal data in a hypothesis-setting randomised controlled trial (two-group plus subgroup covariate design)**.

However, subsequent to this study, Childs et al (2004) [14] performed a randomised controlled trial, in which a treatment modifier effect of the Flynn manipulation prediction rule was demonstrated using a test of subgroup/treatment/time interaction. In this example, the tentative hypothesis regarding treatment effect modifiers was formed in a cohort study and then demonstrated in a subsequent randomised controlled trial, using a test of interaction. In the proposed method framework, these two studies would be categorised as sequential steps in the hypothesis-setting phase. Had the initial study been a randomised controlled trial, the hypothesis formation could have occurred in a single study. The method for such a single study is shown in Figure 5.

The Childs (2004) study examined the treatment modification effect of the entire set of five symptoms and signs in the Flynn manipulation prediction rule. Due to the possibility that a prediction rule derived from a cohort study may contain some predictors that are treatment effect modifiers and some that are prognostic factors, it would be ideal for there to be a mechanism to tease these apart. One way is to perform retrospective (post-hoc) exploratory analysis on the individual symptoms or signs that were included in a randomised controlled trial. Using the Childs (2004) data [14], Fritz et al (2005) [43] did for this one item in the Flynn manipulation prediction rule and showed, using a test of subgroup/treatment interaction, a treatment modifier effect of lumbar spine segmental hypomobility. Theoretically, this post-hoc analysis could be performed on all the prediction rule items to identify which are treatment effect modifiers and further refine the prediction rule.

##### Strengths and weaknesses

An important advantage of subgrouping studies that use supervised statistical techniques to analyse longitudinal data, is that the subgroup has immediate face validity. This is because the subgroup is formed using a clinically relevant dependent (outcome) variable and therefore the clinical utility of the subgroup is readily apparent. A characteristic of supervised techniques is that clinical prediction rules based on the results of such research are usually dependent on a single outcome and therefore this type of research may lead to a proliferation of competing prediction rules. For example, a clinical prediction rule for the outcome of 'return-to-work' may be quite different from a clinical prediction rule for the outcome of 'moderate or more pain', even if the cohort of people, the treatment and the time period of interest are all the same. This is because outcomes such as 'return-to-work' are influenced by other factors than those that are associated with pain reduction, such as the availability of alternative duties or workplace support structures. Similarly, a prediction rule formed when comparing two treatments may not be the same when the comparison treatment is different. In addition, the prediction rule for a monotherapy (such as manipulation) may not hold when that therapy is applied in combination with other treatment (such as manipulation and exercise). Moreover, treatment effects can be time-dependent, and so prediction rules for the same treatment may vary depending on the time period over which participants are studied. Therefore, supervised analysis is likely to result in multiple clinical prediction rules for the same cohort of people and rules that also vary across cohorts of people.

A number of subgrouping studies have used supervised analysis techniques, such as logistic regression, in forms that can only model two subgroups. This is appropriate for modelling dichotomous subgroups, such as responders and non-responders. However, in circumstances where more than two subgroups are to be modelled, other techniques, such as polytomous or multinomial forms of logistic regression, could be used. For example, where a therapy was expensive and had significant side effects, it might be desirable to identify people very likely to respond, people less likely to respond and those very unlikely to respond. In this case, these subgroups could be used to triage people into 'good candidate for this treatment', possible candidate under particular circumstances' and 'poor candidate for this treatment'. Hypothetically, such a therapy in low back pain might be the use of TNF-inhibitor medication for anklyosing spondylitis.

#### Data-driven subgroups from the analysis of cross-sectional data - using 'unsupervised' statistical techniques

One data-driven approach to analysing cross-sectional data for subgroups is the use of 'unsupervised' statistical techniques. As seen earlier, unsupervised techniques do not work backwards from an outcome but are instead used to look for inherent relationships between measurable characteristics in cross-sectional data.

**Figure 6:**
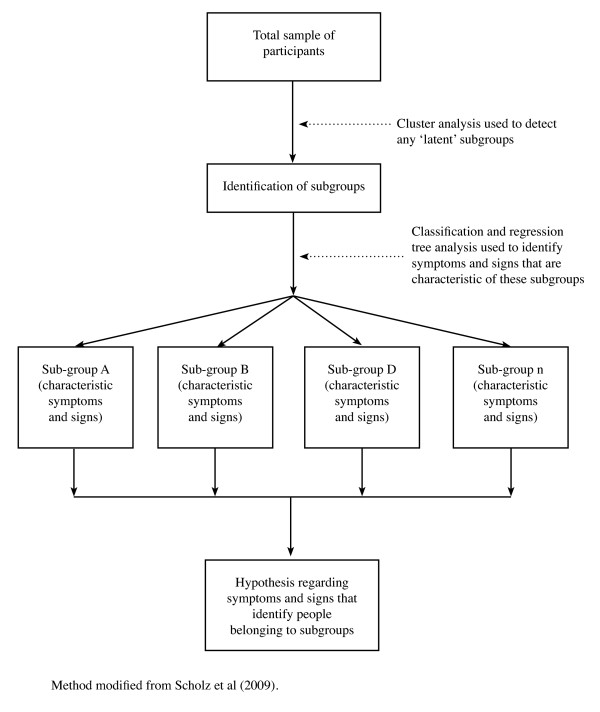
**Example of the use of a 'supervised' statistical technique (cluster analysis) on cross-sectional data in a hypothesis-setting study**.

An example of a subgrouping hypothesis-setting study that used a data-driven unsupervised statistical technique on cross-sectional data is that by Scholtz et al 2009 [44]. In this study of a mixed cohort of people experiencing pain but not necessarily low back pain, hierarchical cluster analysis was used to identify six subgroups of people with neuropathic pain and two subgroups of people with non-neuropathic pain (Figure 6). In a second step, classification tree analysis was used to isolate which symptoms and signs had the greatest discriminatory capacity to classify people into these subgroups. Though not undertaken in this study, the next step within the hypothesis-setting phase of our proposed method framework would be to test these subgroups for treatment modifier or prognostic effects using longitudinal data.

##### Strengths and weaknesses

Unsupervised techniques have some advantages: subgroups detected in this way can later be studied against a range of treatments and outcomes, subgroup formation is not dependent on only one outcome, subgroup formation is not dependent on the efficacy of current treatments, more than two subgroups can be detected in a single analysis, and some unsupervised techniques, such as forms of data-mining, also perform well in the presence of missing data. However, unsupervised techniques are more exploratory than supervised techniques and subgroups are not modelled using a clinical outcome. Therefore, they always require, still within the hypothesis-setting phase, subsequent testing against clinically important outcomes to determine if they are clinically relevant. The major disadvantage of this method is that it is possible, maybe probable, that many subgroups derived using unsupervised techniques have no clinical relevance.

#### Data-driven subgroups from the analysis of cross-sectional data - using 'clinimetric' statistical techniques

Another data-driven method of analysing cross-sectional data for subgroups is the use of 'clinimetric' statistical techniques. In this particular context, this method has been used when researchers seek to detect symptoms and signs that indicate an increased probability that a patient's pain is associated with the presence of a particular pathoanatomic structure (diagnostic subgroup).

**Figure 7:**
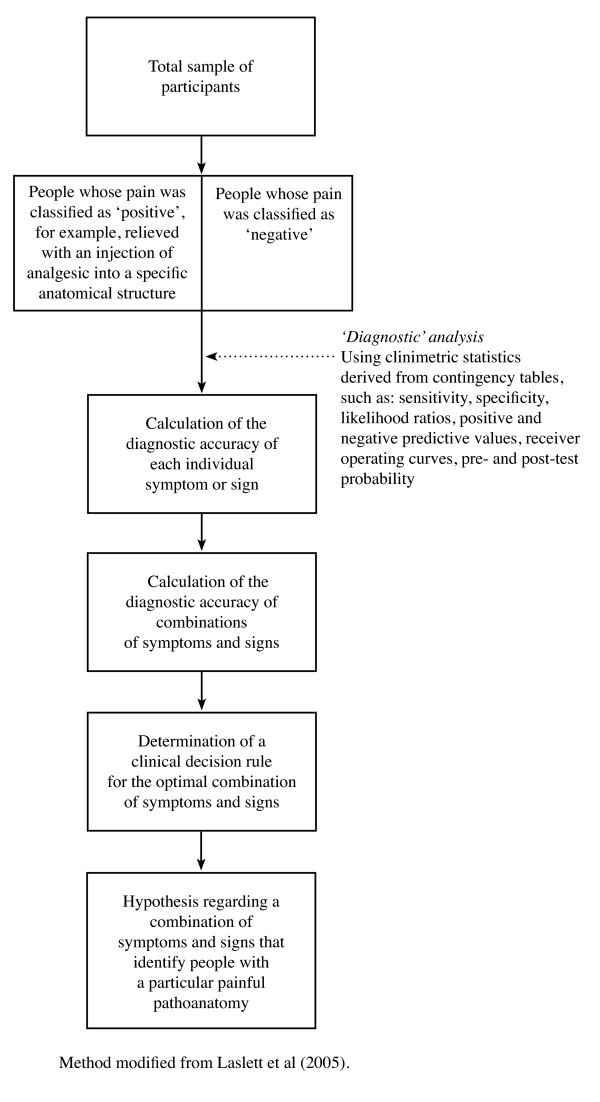
Example of the use of 'clinimetric' statistical techniques (sensitivity and specificity) on cross-sectional data in a 'diagnostic' hypothesis-setting study.

An example of a subgrouping hypothesis-setting study that used data-driven 'clinimetric' statistical techniques on cross-sectional data is Laslett et al 2005 [45]. In this study, people with chronic low back pain seeking a diagnostic evaluation in a radiology clinic, were evaluated using provocative discography and a clinical examination by a skilled physiotherapist. The radiologist and physiotherapist were blind to each other's results and the physiotherapist was blind to previous imaging and injection results. Clinimetric statistics were used to determine the strength of association (diagnostic accuracy) between a positive result on the provocative discography and each of the other symptoms or signs (Figure 7). A clinical decision rule was then formed, consisting of the optimal combination of those symptoms and signs that provided the greatest predictive capacity. Again, though not undertaken in this study, the next step within the hypothesis-setting phase of our proposed method framework would be to test these subgroups for treatment modifier or prognostic effects.

##### Strengths and weaknesses

It is understandable why this 'diagnostic' approach has appeal, as it mimics the Medical Model that has been useful across broad areas of health care. Typically in NSLBP the imaging and clinical findings that have been associated with pathoanatomic structures capable of generating back pain have such a high prevalence in the asymptomatic population that there is considerable uncertainty as to whether they indicate the source of pain in an individual symptomatic patient. Therefore, this 'diagnostic' approach has used the pain response to invasive tests, such as controlled facet injection or provocative discography, to determine the certainty with which a set of clinical symptoms and signs indicate that an individual patient's pain arises from a particular pathoanatomic structure [46-51]. The approach of using pain response to injections in individuals to generalise to populations has been criticised for a number of reasons, including the validity of using response to injection as a reference standard, somatotopic imprecision in the low back, selection bias, spectrum bias, and a lack of concordance in replication studies. However, these criticisms relate more to the construct, design and interpretation of these 'diagnostic' studies than to the clinimetric statistics used.

#### Overfitting, data dredging and sample size

The findings of subgroup research can be erroneous if overfitting or data dredging have occurred. Overfitting is present when the statistical analysis contains too many predictor variables for the size of the dataset. Though differences of opinion exist, some authors have argued that multivariable analysis requires at least 10 outcome events per independent variable to avoid overfitting [52,53]. The number of outcome events is the sum of the occurrences of the outcome of interest in the data. For example, if the outcome of interest were the people who had a very good recovery, and 35% of a cohort of 300 people did recover well, the number of outcome events is 105 (35% of 300) and therefore, approximately 10 independent variables could be simultaneously entered into a multivariable analysis. The presence of overfitting will markedly weaken the probability that the original findings are reproduced in an independent sample.

Data dredging is the search in large data sets for chance findings that are statistically significant and their reporting without testing if they are spurious associations through replication in an independent data set. Overfitting and data dredging reinforce the desirability of independent sample replication before subgroup predictors are given credence. This may prevent the clinical application or further fruitless testing of chance findings. Despite the importance of this step, a recent systematic review of predictors of chronicity in NSLBP found that, depending on the outcome measure used, only 1 in 12 to 1 in 30 included studies had tested their multivariable findings in an independent sample [54].

All statistical methods for subgroup research require larger sample sizes than studies that are powered to detect effects observable in a whole group. For example, two-group randomised controlled trials designed to quantify the impact of treatment effect modifiers, require approximately four times the sample size of a conventional controlled trial powered to detect a main effect of the same size [55]. Similarly, the variability present in NSLBP is likely to warrant larger sample sizes in cohort studies and 'diagnostic' studies, than in conditions where the link between pain and pathology is stronger. Hancock et al (2009) [23] have argued that estimates of treatment effect modification require narrow confidence intervals to be convincing. Narrower confidence intervals, whether around point estimates of treatment effect modification, prognostic risk or diagnostic accuracy, are measures of increased certainty. Narrower confidence intervals allow increased confidence in inferences about clinically important subgroups but do not preclude the need for validation studies.

#### The need for all phases of subgrouping research

All the research designs and statistical techniques shown in the method pathways in Figures 2 and 3 have their advocates and detractors. The perfect study has not been conducted, as methods are constantly evolving. However, if we are to determine whether subgroup-tailored treatment or generic treatment is better clinical practice, imperfect hypothesis-setting studies will need to be tolerated in the knowledge that further testing is required in a rigorous hypothesis-testing phase and subsequent validation phases. Regardless of the methods used to form subgroup hypotheses, whether these hypotheses concern prognostic effects or treatment effects, there is a need to continue through the other phases of subgrouping research to determine whether these effects are reproducible, generalisable and of clinical importance. Erroneous subgroup findings will not survive the challenge of these later phases of investigation.

We propose that a commitment to the rigor implicit in the method framework is a standard that all proponents of subgroups should meet if subgroup hypotheses are to have scientific credibility. Similarly, it could be argued that subgroup findings that are still in the hypothesis-setting stage are premature to market to clinicians, due to the high probability of spurious findings.

Even where subgrouping findings have shown acceptable reproducibility, generalisability and important effect size, it will take appropriately-designed impact studies to demonstrate whether subgrouping does change practice and improve outcomes in routine care settings. Only where all these criteria are satisfied can evidence-based clinical guidelines confidently recommend subgrouping for routine care.

## Summary

There is a need for a method framework for subgrouping studies in low back pain due to considerable interest in subgrouping and clinical prediction rules, a proliferation of research methods, and variability in how subgroup results are interpreted. The method framework presented in this article is not prescriptive but is presented to further conversation amongst researchers and clinicians about a suitable roadmap with which to coherently plan, stage, perform and evaluate subgrouping research. The studies used as examples in this method framework were from low back pain research but the methods are equally applicable to neck pain and may also be applicable to other musculoskeletal conditions. The suggested framework provides a platform for modification and extension by the research community, and needs to be regularly updated as method evolves.

## Competing interests

The manuscript submitted does not contain information about medical devices or drugs. No benefits in any form have been, or will be, received from a commercial party related directly or indirectly to the subject of this manuscript.

## Authors' contributions

The conception and design of the paper was by PMK. All authors (PMK, JLK, CLY) were involved in the analysis and interpretation of data, drafting and revision of the manuscript, and gave final approval of the manuscript.

## Pre-publication history

The pre-publication history for this paper can be accessed here:

http://www.biomedcentral.com/1471-2288/10/62/prepub

## Supplementary Material

Additional file 1 **A method for constructing a clinical prediction rule**. Click here for file

## References

[B1] Deyo R Rainville J Kent D What can the history and physical examination tell us about low back pain? JAMA 1992 10 760 765 10.1001/jama.268.6.760 1386391

[B2] Spengler DM David DP Wiesel SW Industrial low back pain: A practical approach Industrial low back pain: A comprehensive approach 1985 Charlottesville, VA, USA: The Michie Company 869 871

[B3] Kent P Keating JL Buchbinder R Searching for a conceptual framework for nonspecific low back pain Man Ther 2009 10 387 396 10.1016/j.math.2008.07.003 18793868

[B4] Kent PM Keating J Do primary-care clinicians think that non-specific low back pain is one condition? Spine 2004 10 1022 1031 10.1097/00007632-200405010-00015 15105677

[B5] Borkan J Koes B Reis S Cherkin D A report from the Second International Forum for primary care research on low back pain - Reexamining priorities Spine 1998 10 1992 1996 10.1097/00007632-199809150-00016 9779533

[B6] Delitto A Erhard RE Bowling RW A treatment-based classification approach to low back syndrome: identifying and staging patients for conservative treatment Phys Ther 1995 10 470 489 7770494 10.1093/ptj/75.6.470

[B7] Long A Donelson R Fung T Does it matter which exercise? A randomized control trial of exercise for low back pain Spine 2004 10 2593 2602 10.1097/01.brs.0000146464.23007.2a 15564907

[B8] Petersen T Thorsen H Manniche C Classification of non-specific low back pain: a review of the literature on classifications systems relevant to physiotherapy Physical Therapy Reviews 1999 10 265 281

[B9] O'Sullivan P Classification of lumbopelvic pain disorders--Why is it essential for management Man Ther 2006 10 169 170 10.1016/j.math.2006.01.002 16977708

[B10] Hill JC Dunn KM Lewis M Mullis R Main C Foster NE Hay EM A primary care back pain screening tool: Identifying patient subgroups for initial treatment Arthritis Rheumat 2008 10 632 641 10.1002/art.23563 18438893

[B11] Laslett M McDonald B Troop H Aprill CN Oberg B Strength of agreement between diagnosis reached by clinical examination and available reference standards: A prospective validity study of 216 patients with lumbopelvic pain and/or symptoms referred into the lower extremity BMC J Musculoskel Dis 2005 10 doi: 10.1186/1471-2474-1186-1128 10.1186/1471-2474-6-28 PMC1184083 15943873

[B12] Petersen T Laslett M Thorsen H Manniche C Ekdahl C Jacobsen S Diagnostic classification of non-specific low back pain. A new system integrating patho-anatomic and clinical categories Physiother Theory Pract 2003 10 213 237

[B13] Brennan GP Fritz JM Hunter SJ Thackeray A Delitto A Erhard RE Identifying subgroups of patients with acute/subacute "nonspecific" low back pain - Results of a randomized clinical trial Spine 2006 10 623 631 10.1097/01.brs.0000202807.72292.a8 16540864

[B14] Childs JD Fritz JM Flynn TW Irrgang JJ Johnson KK Majkowski GR Delitto A A clinical prediction rule to identify patients with low back pain most likely to benefit from spinal manipulation: A validation study Ann Int Med 2004 10 920 928 15611489 10.7326/0003-4819-141-12-200412210-00008

[B15] Hicks GE Fritz JM Delitto A McGill SM Preliminary development of a clinical prediction rule for determining which patients with low back pain will respond to a stabilization exercise program Arch Phys Med Rehab 2005 10 1753 1762 10.1016/j.apmr.2005.03.033 16181938

[B16] Kent PM Keating JL Classification in non-specific low back pain - what methods do primary care clinicians currently use? Spine 2005 10 1433 1440 10.1097/01.brs.0000166523.84016.4b 15959374

[B17] McGinn TG Guyatt GH Wyer PC Naylor CD Stiell IG Richardson WS Users' guides to the medical literature: XXII: how to use articles about clinical decision rules JAMA 2000 10 79 84 10.1001/jama.284.1.79 10872017

[B18] Altman DG Vergouwe Y Royston P Moons KG Prognosis and prognostic research: validating a prognostic model BMC 2009 10 b605 10.1136/bmj.b605 19477892

[B19] Rothwell P Subgroup analysis in randomised controlled trials: importance, indications, and interpretation Lancet 2005 10 176 186 10.1016/S0140-6736(05)17709-5 15639301

[B20] Royston P Moons KG Altman DG Vergouwe Y Prognosis and prognostic research: developing a prognostic model BMJ 2009 10 b604 10.1136/bmj.b604 19336487

[B21] Wittes J On looking at subgroups: Editorial Circulation 2009 10 912 915 10.1161/CIRCULATIONAHA.108.836601 19237669

[B22] Yusuf S Wittes J Probstfield J Tyroler HA Analysis and interpretation of treatment effects in subgroups of patients in randomized clinical trials JAMA 1991 10 93 98 10.1001/jama.266.1.93 2046134

[B23] Hancock M Herbert R Maher CG A guide to interpretation of studies investigating subgroups of responders to physical therapy interventions Phys Ther 2009 10 698 704 10.2522/ptj.20080351 19465372

[B24] Klebanoff MA Subgroup analysis in obstetrics clinical trials Am J Obstet Gynec 2007 10 119 122 10.1016/j.ajog.2007.02.030 17689621

[B25] Hayden JA Côté P Steenstra IA Bombardier C Group ftQ-LW Identifying phases of investigation helps planning, appraising and applying the results of explanatory prognosis studies J Clin Epi 2008 in press 10.1016/j.jclinepi.2007.08.005 18471659

[B26] Moons KG Royston P Vergouwe Y Grobbee DE Altman DG Prognosis and prognostic research: what, why and how? BMC 2009 10 b375 10.1136/bmj.b375 19237405

[B27] Hancock MJ Maher CG Latimer J Herbert RD McAuley JH Independent evaluation of a clinical prediction rule for spinal manipulative therapy: a randomised controlled trial Euro Spine J 2008 10 936 943 10.1007/s00586-008-0679-9 PMC2443269 18427840

[B28] Kent P Hancock M Petersen DH Mjøsund HJ Choosing appropriate study designs for particular questions about treatment subgroups Journal of Manual and Manipulative Therapy 2010 in press accepted 16 March 2010 10.1179/106698110X12640740712419 PMC3109682 21886425

[B29] Kent P Mjøsund HL Petersen DH Does targeting manual therapy and/or exercise improve patient outcomes in nonspecific low back pain? - A systematic review BMC Medicine 2010 10 22 doi:10.1186/1741-7015-8-22 10.1186/1741-7015-8-22 20377854 PMC2873245

[B30] Pocock SJ Assmann SE Enos LE Kasten LE Subgroup analysis, covariate adjustment and baseline comparisons in clinical trial reporting: current practice and problems Stat Med 2002 10 2917 2930 10.1002/sim.1296 12325108

[B31] Beattie P Nelson RM Clinical prediction rules: what are they and what do they tell us? Aust Journal Physio 2006 10 157 163 10.1016/s0004-9514(06)70024-1 16942450

[B32] Bland JM Altman DG Statistical methods for assessing agreement between two methods of clinical measurement Lancet 1986 307 310 2868172

[B33] Bombardier C Tugwell P Methodological considerations in functional assessment J Rheumat 1987 10 6 10 3498841

[B34] Kirsner B Guyatt G Methodological framework for assessing health indices J Chronic Dis 1985 10 27 36 10.1016/0021-9681(85)90005-0 3972947

[B35] Saal FE Downey RG Lahey MA Rating the ratings: Assessing the psychometric quality of rating data Psychol Bull 1980 10 413 428 10.1037/0033-2909.88.2.413

[B36] Uebersax JS Diversity of decision making models and the measurement of interrater agreement Psychol Bull 1987 10 140 146 10.1037/0033-2909.101.1.140

[B37] Ferreira PH Ferreira ML Hodges PW Changes in recruitment of the abdominal muscles in people with low back pain ultrasound measurement of muscle activity Spine 2004 10 2560 2566 10.1097/01.brs.0000144410.89182.f9 15543074

[B38] Laupacis A Sekar N Stiell IG Clinical prediction rules. A review and suggested modifications of methodological standards JAMA 1997 10 488 494 10.1001/jama.277.6.488 9020274

[B39] Cameron C Naylor CD No impact from active dissemination of the Ottawa Ankle Rules: Further evidence of the need for local implementation of practice guidelines Can Med Assoc J 1999 10 1165 1168 10234347 PMC1230269

[B40] McKenzie R Prophylaxis in recurrent low back pain N Z Med J 1979 10 22 23 155230

[B41] O'Sullivan PB Beales DJ Diagnosis and classification of pelvic girdle pain disorders--Part 1: A mechanism based approach within a biopsychosocial framework Man Ther 2007 10 86 97 10.1016/j.math.2007.02.001 17449432

[B42] Flynn T Fritz JW Whitman M Wainner RS Magel J Rendeiro D Butler B Garber M Allison S A clinical prediction rule for classifying patients with low back pain who demonstrate short-term improvement with spinal manipulation Spine 2002 10 2835 2843 10.1097/00007632-200212150-00021 12486357

[B43] Fritz JM Whitman JM Childs JD Lumbar spine segmental mobility assessment: An examination of validity for determining intervention strategies in patients with low back pain Arch Phys Med Rehab 2005 10 1745 1752 10.1016/j.apmr.2005.03.028 16181937

[B44] Scholz J Mannion RJ Hord DE Griffin RS Rawal B Zheng H Scoffings D Phillips A Guo J Laing JC A novel tool for the assessment of pain: Validation in low back pain PLoS Med 2009 10 e1000047 10.1371/journal.pmed.1000047 19360087 PMC2661253

[B45] Laslett M Oberg B Aprill CN McDonald B Centralization as a predictor of provocation discography results in chronic back pain, and the influence of disability and distress on diagnostic power The Spine Journal 2005 10 370 380 10.1016/j.spinee.2004.11.007 15996606

[B46] Donelson R Aprill C Medcalf R Grant W A prospective study of centralization of lumbar and referred pain: A predictor of symptomatic discs and annular competence Spine 1997 10 1115 1122 10.1097/00007632-199705150-00011 9160470

[B47] Laslett M Young SB Aprill CN McDonald B Diagnosing painful sacroiliac joints: A validity study of a McKenzie evaluation and sacroiliac provocation tests Aust J Physiother 2003 10 89 97 12775204 10.1016/s0004-9514(14)60125-2

[B48] Laslett M Diagnostic accuracy of the clinical examination compared to available referenve standards in chronic low back pain patients PhD 2005 Linkopings Universitet, Division of Physiotherapy

[B49] Manchikanti L Pampati V Fellows B Baha A The inability of the clinical picture to characterize pain from the facet joints Pain Physician 2000 10 158 166 16906195

[B50] Schwarzer A Aprill C Bogduk N The sacroiliac joint in chronic low back pain Spine 1995 10 31 37 10.1097/00007632-199501000-00007 7709277

[B51] Young S Aprill C Laslett M Correlation of clinical examination characteristics with three sources of chronic low back pain The Spine Journal 2003 10 460 465 10.1016/S1529-9430(03)00151-7 14609690

[B52] Concato J Feinstein AR Holford TR The risk of determining risk with multivariate models Ann Int Med 1993 10 201 210 8417638 10.7326/0003-4819-118-3-199302010-00009

[B53] Peduzzi P Concato J Kemper E Holford TR Feinstein AR A simulation study of the number of events per variable in logistic regression analysis J Clin Epidemiol 1996 10 1373 1379 10.1016/S0895-4356(96)00236-3 8970487

[B54] Kent PM Keating JL Can we predict poor recovery from recent-onset nonspecific low back pain? A systematic review Man Ther 2008 10 12 28 10.1016/j.math.2007.05.009 17658288

[B55] Brookes ST Whitely E Egger M Smith GD Mulheran PA Peters TJ Subgroup analyses in randomized trials: risks of subgroup-specific analyses: power and sample size for the interaction test Clin Epi 2004 10 229 236 10.1016/j.jclinepi.2003.08.009 15066682

