# Supplementary material for: Research methods for subgrouping low back pain
Source: BMC Med Res Methodol. 2010 Jul 3;10:62. doi: 10.1186/1471-2288-10-62 (PMC2908106; doi:10.1186/1471-2288-10-62)
Supplement: Additional file 1 — A method for constructing a clinical prediction rule. [file 1471-2288-10-62-S1.DOC]

***Additional file 1. A method for constructing a clinical prediction rule***

Clinical prediction rules enable the implications of a sub-grouping system to be expressed in clinical interpretable ways. They also allow the accuracy of this predictive capacity to be described. An example of a clinical prediction rule in NSLBP is “a person with any 4 of 5 (specified) symptoms or signs has a 95% probability of responding to spinal manipulation (sensitivity 63%, specificity 97%, positive post-test probability 95%)” [1]. Such prediction rules are readily understood by clinicians and there is evidence from other areas of health care that these kinds of prediction rules can change clinical practice and improve health system outcomes [2-5]. The creation of a decision rule usually involves the use of contingency tables (see Table 1).

Table 1: An example of a contingency table.

|  | Improved with treatment | Did not improve with treatment | Totals |
| --- | --- | --- | --- |
| Had symptom or sign | 463  True positive (A) | 31  False positive (B) | 494 |
| Did not have symptom or sign | 78  False negative (C) | 569  True negative (D) | 647 |
| Totals | 541 | 600 | 1141 |

Contingency tables are a statistical device that require both the predictor and the outcome to be one of two events, that is, dichotomous. Predictor variables measured using categorical or interval scales are often retained in their original metric during bivariate and multivariate modelling, rather than being dichotomised, as this retains more information about the relationship. Such variables, if retained in multi-variable modelling, can be dichotomised post-hoc. This post-hoc dichotomisation often uses receiver operating characteristic (ROC) curve analysis to determine the cut-point at which the division produces two conditions that most accurately enable prediction of outcome. Some statistical techniques, such as data-mining or classification and regression tree analysis automatically determine optimal cut-points.

Following this dichotomisation, the number of positive predictor variables present for each participant can then be counted. For example, if there were five symptoms or signs retained in the predictive model, the number of positive predictor variables for each participant would range from 0 to 5. These figures are then tabulated to show how many people had a particular number of predictor variables present and of them, the number of those people who had a positive or negative outcome (shown in Table 2, using data from Flynn et al 2002 [1]).

Table 2: Example of a tabulation of those participants whose positive number of predictor variables were at each level of a prediction rule (data from Flynn et al 2002).

| Number of variables positive | Number of people who responded to the treatment | Number of people who did not respond to the treatment |
| --- | --- | --- |
| 5 | 6 | 0 |
| 4 | 14 | 1 |
| 3 | 10 | 13 |
| 2 | 2 | 19 |
| 1 | 0 | 5 |
| 0 | 0 | 1 |

These numbers can then be used to construct contingency tables and clinimetrics, such as sensitivity, specificity, likelihood ratios and probabilities are calculated for this prediction rule (shown in Table 3, again using data extracted from Flynn et al 2002[1]).

Table 3: The contingency table and clinimetrics for a clinical prediction rule, for people with different numbers of positive predictor symptoms or signs (data extracted from Flynn et al 2002).

| **Number of positive predictor variables (symptoms or signs)** | 1  or more | 2  or more | 3  or more | 4  or more | 5 |
| --- | --- | --- | --- | --- | --- |
| **A** (true positive) | 32 | 32 | 30 | 20 | 6 |
| **B** (false positive) | 38 | 33 | 14 | 1 | 0 |
| **C** (false negative) | 0 | 0 | 2 | 12 | 26 |
| **D** (true negative) | 1 | 6 | 25 | 38 | 39 |
| **Sensitivity**  (95% CI) | 100%  (89%,100%) | 100%  (89%,100%) | 94%  (80%,98%) | 63%  (45%,77%) | 19%  (9%,35%) |
| **Specificity**  (95% CI) | 3%  (1%,13%) | 15%  (7%,30%) | 64%  (48%,77%) | 97%  (87%,100%) | 100%  (91%,100%) |
| **Positive likelihood ratio***  (95% CI) | 1.03  (0.98,1.08) | 1.18  (1.03,1.35) | 2.61  (1.70,4.10) | 24.38  (3.46,171.88) | infinity  (2.02,infinity) |
| **Pre-test probability of improvement** | 45% | | | | |
| **Positive post-test probability of improvement***  (95% CI) | 46%  (35%, 58%) | 49%  (37%, 61%) | 68%  (54%, 82%) | 95%  (86%, 100%) | _ |

*Both positive and negative likelihood ratios and/or post-test probabilities can be tabulated

95%CI = 95% confidence interval

Usually, the final step in this process is to determine an optimal number of predictor variables for the clinical prediction rule. In this example, Flynn et al (2002) chose four or more variables. Choosing a single cut-point such as this makes the rule easier to explain and to remember. However, if the prediction rule is used electronically, the complete table can be embedded in a computer algorithm to provide patient-specific estimates of treatment response based on the number of positive predictor variables that the patient displayed.

**References**

1. Flynn T, Fritz JW, Whitman M, Wainner RS, Magel J, Rendeiro D, Butler B, Garber M, Allison S: **A clinical prediction rule for classifying patients with low back pain who demonstrate short-term improvement with spinal manipulation**. *Spine* 2002, **27**(24):2835-2843.

2. Auleley GR, Ravaud P, Giraudeau BE, Kerboull L, Nizard R, Massin P, Garreau de Loubresse C, Vallee C, Durieux P: **Implementation of the Ottawa ankle rules in France: a multicenter randomised controlled trial**. *JAMA* 1997, **277**:1935-1939.

3. Nichol G, Steill IG, G.A. W, Juergensen LS, Laupacis A: **An economic analysis of the Ottawa knee rule**. *Ann Emerg Med* 1999, **34 (4 pt1)**:438-447.

4. Stiell IG, McKnight RD, G.H. G, McDowell I, Nair RC, Wells GA, Johns C, Worthington JR: **Implementation of the Ottawa ankle rules**. *JAMA* 1994, **271**:827-832.

5. Verbeek PR, Stiell IG, Hebert G, Sellens C: **Ankle radiographs after learning a decision rule: a 12-month follow-up**. *Acad Emerg Med* 1997, **4**:776-779.
